# Impact of Gut Microbiota on the Clinical Course and Treatment Outcomes of Colorectal Cancer—A Systematic Review

**DOI:** 10.3390/medicina62061050

**Published:** 2026-05-28

**Authors:** Ilektra Kyrochristou, Fotios Fousekis, Gerasimia D. Kyrochristou, Dimitrios Schizas, George Pappas-Gogos, Dimitrios Raptis, Orestis Ioannidis, Konstantinos Vlachos, Georgios D. Lianos

**Affiliations:** 1 Department of Surgery, University Hospital of Ioannina, 45500 Ioannina, Greece; ersie.cd@gmail.com (G.D.K.); pappasg8@gmail.com (G.P.-G.); vlachoskonstantinos@yahoo.gr (K.V.); glianos@uoi.gr (G.D.L.); 2 2nd Department of Surgery, General Hospital of Nikaia and Piraeus, 18454 Piraeus, Greece; 3 Department of Gastroenterology, University Hospital of Ioannina, 45500 Ioannina, Greece; fotisfous@gmail.com; 4 1st Department of Surgery, National and Kapodistrian University of Athens, Laikon General Hospital, 11527 Athens, Greece; schizasad@gmail.com; 5 2nd Department of Surgery, Aristotle University of Thessaloniki, 54124 Thessaloniki, Greece; 6 4th Department of Surgery, Aristotle University of Thessaloniki, 54124 Thessaloniki, Greece; orestisioannidis@gmail.com

**Keywords:** gut microbiota, microbiome, colorectal cancer, taxonomic research, *Fusobacterium nucleatum*

## Abstract

*Background and Perspectives:* As colorectal cancer research focuses on improving screening policies and treatment strategies, the gut microbiome is emerging as a novel diagnostic and prognostic biomarker. This systematic review aims to present the available data on the role of gut microbiota in colorectal cancer diagnosis, prognosis, and treatment response. *Materials and Methods:* A systematic search under the PRISMA recommendation was conducted in PubMed database, until February 2026. Original human studies evaluating associations between gut microbiome composition and CRC diagnosis, survival outcomes, or therapeutic response were included. Both stool- and tissue-based analyses were considered. A qualitative synthesis of the data was performed. *Results:* Thirty-six studies met the inclusion criteria, encompassing case–control cohorts, prospective survival analyses, and early-phase translational trials. Across populations and sequencing methodologies, gut microbiome alterations were consistently identified, with enrichment of oral-derived anaerobes, particularly *Fusobacterium nucleatum*, and depletion of beneficial commensal taxa in CRC patients compared with controls. Beta-diversity analyses frequently showed distinct clustering of microbial communities between the CRC and control groups, whereas alpha-diversity findings were heterogeneous. Several stool-based multi-species classifiers demonstrated good to excellent diagnostic performance, particularly when combined with established screening modalities. Tumor-associated microbial signatures were further associated with adverse survival outcomes and, in exploratory cohorts, with differential treatment response. Emerging evidence suggests that the microbiome may represent a modifiable environmental factor, particularly relevant in early-onset CRC. *Conclusions:* The gut microbiome represents a promising adjunctive biomarker for CRC diagnosis and prognostic stratification, with potential implications for precision oncology. However, methodological heterogeneity and the need for prospective validation currently limit its routine clinical implementation.

## 1. Introduction

As of 2025, colorectal cancer (CRC) remains a major global health burden, ranking as the third most commonly diagnosed malignancy and the second leading cause of cancer-related mortality worldwide [1]. According to the World Health Organization, approximately 1.9–2 million new cases are diagnosed each year [2]. Notably, the increasing incidence of CRC among individuals younger than 50 years has raised significant concern, highlighting potential gaps in current screening strategies and the growing influence of environmental and lifestyle-related factors [3].

In recent years, the human gut microbiome has emerged as a critical component of host physiology and disease. Rather than being viewed as a passive microbial community, it is now increasingly recognized as a dynamic “organ-like” system that interacts with the host through metabolic, immunological, and signaling pathways [4]. Its role in cancer biology extends beyond carcinogenesis and includes potential influences on tumor progression, immune modulation, and response to therapy [5,6,7,8,9].

A substantial body of experimental and translational research has demonstrated that alterations in gut microbiota composition—commonly referred to as dysbiosis—may contribute to colorectal carcinogenesis through mechanisms such as chronic inflammation, production of microbial genotoxins, disruption of epithelial barrier integrity, and modulation of oncogenic signaling pathways [10,11,12,13,14,15,16,17]. Importantly, microbial changes have been observed not only in established CRC but also during the adenoma–carcinoma sequence, suggesting a potential role in early tumorigenesis and disease progression [18].

Despite this growing evidence, the clinical relevance of the gut microbiome in CRC remains incompletely defined. Existing studies exhibit considerable heterogeneity across patient populations, geographic regions, sequencing methodologies, and analytical pipelines [10,11,12,13]. While this variability complicates direct comparisons, it also reflects the complex and context-dependent nature of host–microbiome interactions. Importantly, consistent patterns—such as the enrichment of specific oral-derived anaerobes and depletion of beneficial commensals—have been reproduced across multiple independent cohorts, supporting the biological validity of microbiome alterations in CRC [18].

Furthermore, unlike static genetic alterations, the gut microbiome represents a potentially modifiable factor influenced by diet, medications, and environmental exposures [11,12,13]. This raises the possibility that microbiome-based interventions could contribute not only to improved diagnostic and prognostic strategies but also to preventive and therapeutic approaches, particularly in the context of early-onset CRC.

Therefore, despite methodological heterogeneity, synthesizing the available clinical evidence remains essential. This systematic review critically evaluates current human studies investigating the role of the gut microbiome in CRC diagnosis, prognosis, and treatment response, while highlighting limitations, sources of variability, and future directions for research and clinical application.

## 2. Materials and Methods

**Figure 1:**
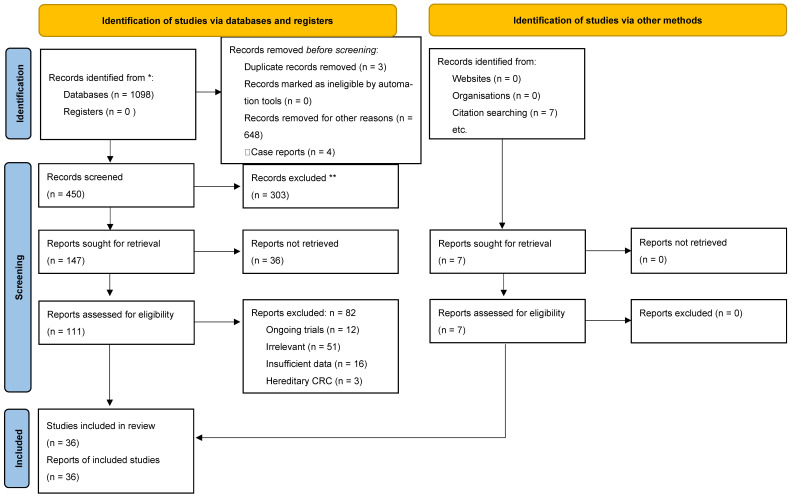
PRISMA 2020 flow diagram for new systematic reviews which included searches of databases, registers and other sources. * PUBMED ** All records were excluded by a human.

A systematic search has been conducted in PubMed database, for the period 1 January 2006–22 February 2026, under the following algorithm: (colorectal cancer OR colon cancer OR rectal cancer OR colorectal neoplas* OR CRC) AND (microbiome OR microbiota OR metagenom* OR 16S OR “shotgun sequencing” OR “fecal” OR “stool” OR “gut”) AND (diversity OR “alpha diversity” OR “beta diversity” OR “Shannon” OR “Simpson” OR “UniFrac” OR Bray OR Jaccard OR PERMANOVA OR “differential abundance” OR pathway OR MetaPhlAn OR HUMAnN) AND (survival OR OS OR DFS OR PFS OR recurren* OR stage OR “treatment response” OR RECIST OR pCR OR “chemoradiation” OR immunotherap*). The study selection process is summarized in a PRISMA 2020 flow diagram (Figure 1), including identification, screening, eligibility, and inclusion phases. This review was not prospectively registered.

Of the 1098 results, cohort studies (prospective/retrospective), nested case–control studies, clinical trials with prognostic analyses, and biobank-based analyses were included in the synthesis. Case reports/series (<10 patients), editorials, pediatric studies, experimental/animal studies, narrative reviews, and studies in languages other than English were excluded. Two independent reviewers screened titles and abstracts for eligibility. Full-text articles were subsequently assessed for inclusion. Disagreements were resolved by consensus or consultation with a third reviewer.

Finally, 29 studies were included in the synthesis, and via the snowball technique, seven additional studies were identified from their references and incorporated into the qualitative analysis. The PubMed algorithm and its results are shown in Figure 1. The review was designed and conducted in accordance with the PRISMA 2020 guidelines [19].

Data extracted included: study design, sample size, patient characteristics, tumor stage, treatment modality, microbiome analysis method (16S rRNA sequencing, shotgun metagenomics), diversity metrics, differential taxa, clinical outcomes (OS, DFS, PFS), and main conclusions.

Risk of bias was assessed using the Newcastle–Ottawa Scale (NOS) for cohort and case–control studies. Non-randomized interventional and treatment-response studies were evaluated using the ROBINS-I tool. Risk of bias assessment was performed independently by two reviewers, with disagreements resolved by consensus. The population under investigation comprised adult patients with colorectal cancer, and the main research objective was to assess the gut microbiome in stool, tissue, or mucosal samples using culture-independent methods (16S rRNA, shotgun metagenomics, qPCR) or targeted assays. Primary outcomes were categorized into three categories:

1. Diagnostic value of specific microbial signatures in the detection of CRC.
2. Prognostic role of microbial agents in terms of Overall Survival (OS), Disease-Free Survival (DFS), and Recurrence Rate (RR).
3. Measurement of response to treatment, in groups of patients where either therapeutic surgery or adjuvant treatment has taken place.

Secondary outcomes included differences in microbiome composition across populations and the potential influence of sampling and identification processes (e.g., 16S rRNA vs. shotgun metagenomics) on the identified microbes.

Due to heterogeneity in microbiome methodologies and outcome reporting, a meta-analysis was not feasible; therefore, a qualitative synthesis was performed.

## 3. Results

Overall, 36 studies were included in the synthesis, totaling 48,927 individuals and 22,716 CRC patients (Table 1) [20,21,22,23,24,25,26,27,28,29,30,31,32,33,34,35,36,37,38,39,40,41,42,43,44,45,46,47,48,49,50,51,52,53,54,55]. Patients were recruited from all five continents. Their mean age ranged from 52.4 to 76.75 years, and their mean BMIs ranged from 22.1 to 32.7 kg/m^2^. Sex distribution could not be consistently extracted, as it was not reported in the majority of studies. Patients’ demographics and disease stage are presented in Table 2.

Case–control (majority) studies, prospective and retrospective cohorts, clinical trials, Meta-analyses, and Mendelian randomization studies were included in the synthesis. The microbiome research used stool and/or tissue samples, and the DNA sequencing methods were 16S rRNA and shotgun metagenomics. Full information on the extraction, sequencing, and platform methods is available in Supplementary Table S2.

Most included studies demonstrated moderate methodological quality, as assessed by the Newcastle–Ottawa Scale and the ROBINS-I. The primary sources of bias included inadequate adjustment for confounding factors (e.g., antibiotic exposure, diet, tumor stage), heterogeneity in microbiome sampling and sequencing methodologies, and limited external validation of findings. No study was classified as having a critical risk of bias (Supplementary Table S1).

### 3.1. Diagnostic Value of Gut Microbiome in CRC

Across the included studies (*n* = 36), the majority (approximately 24/36, ~67%) evaluated the diagnostic potential of gut microbiome alterations using stool and/or tissue samples [20,21,22,23,24,27,28,31,32,33,39,41,45,50,51,52,53,54,55]. Despite differences in study design, geographic origin, and sequencing methodologies, most studies consistently demonstrated significant alterations in microbial composition in patients with colorectal cancer compared with controls, supporting the reproducibility of CRC-associated dysbiosis [23,28,32,33].

At the taxonomic level, enrichment of oral-derived anaerobic species—particularly *Fusobacterium nucleatum*, *Parvimonas micra*, and *Peptostreptococcus stomatis*—was among the most consistent findings. *Fusobacterium nucleatum* was reported to be significantly enriched in CRC patients in the majority of studies assessing species-level composition (approximately 10–12 studies, depending on the detection method), across both stool- and tissue-based analyses [25,31,39,53,54]. Similarly, *Parvimonas micra* and *Peptostreptococcus stomatis* were identified as part of CRC-associated microbial signatures in multiple independent cohorts, particularly in studies employing shotgun metagenomic sequencing and the development of multi-species classifiers [39,53].

In contrast, several studies reported depletion of short-chain fatty acid–producing commensal bacteria, including butyrate-producing taxa, in CRC patients (reported in approximately 6–8 studies), supporting a shift toward a pro-inflammatory and metabolically altered microbial environment [24,33].

Importantly, individual taxa demonstrated only moderate discriminatory capacity when evaluated independently [39]. In contrast, multi-species microbial panels consistently outperformed single-organism biomarkers. Approximately 8–10 studies developed and validated multi-species classifiers, most of which achieved area under the receiver operating characteristic curve (AUC) values ranging from 0.80 to 0.90 [20,23,32,33,41,54]. These findings were particularly robust in shotgun metagenomic studies, whereas 16S rRNA-based approaches showed greater variability in classification performance [23,32,45,54]. This suggests that CRC-associated dysbiosis is multifactorial and is better captured by composite microbial signatures than by single taxa.

Regarding ecological diversity metrics, findings were heterogeneous. Reduced alpha diversity (e.g., Shannon index) in CRC patients was reported in approximately 5–6 studies, primarily in stool-based metagenomic analyses [33,51,53]. However, a comparable number of studies reported no significant differences in alpha diversity between CRC and control groups [21,28,41], suggesting inconsistent results across cohorts. These discrepancies appeared to be influenced by differences in sequencing depth, analytical pipelines, and population characteristics.

In contrast, beta-diversity analyses yielded more consistent results. Approximately 10–12 studies reported significant separation between CRC and control groups based on community composition using metrics such as Bray–Curtis dissimilarity and UniFrac distances [27,31,33,51,53,54]. These findings were observed across both stool- and tissue-based studies and across sequencing platforms, suggesting that microbial community structure, rather than overall diversity magnitude, may be more diagnostically informative.

Sensitivity and specificity varied across studies, with generally moderate-to-high specificity and more variable sensitivity. Notably, 3–4 studies demonstrated that combining microbiome-based classifiers with established screening modalities, such as fecal occult blood testing (FOBT), improved diagnostic performance, particularly sensitivity [41,54]. This supports the concept that microbiome profiling may serve as an adjunct rather than a replacement for current screening strategies.

However, cross-cohort validation studies highlighted important limitations. Although several taxa were reproducibly associated with CRC, model performance often declined when classifiers were applied to external populations [20,23,32,45]. This variability likely reflects differences in diet, ethnicity, environmental exposures, and technical methodologies, underscoring the need for standardized protocols and large-scale validation.

These findings are further synthesized in Table 3 and Table 4, which summarize microbial signatures and their diagnostic performance across studies.

Overall, current evidence supports the gut microbiome as a promising non-invasive diagnostic biomarker for colorectal cancer. The strongest and most consistent evidence derives from multi-species stool-based classifiers validated across independent cohorts. Nevertheless, methodological heterogeneity and limited cross-population generalizability remain key barriers to clinical implementation.

### 3.2. Microbiome and CRC Prognosis

Seven cohort studies evaluated the association between tumor-associated microbiota and long-term outcomes in colorectal cancer. Most analyses were based on tumor tissue using qPCR or 16S rRNA sequencing, and the primary endpoints included overall survival (OS), colorectal-cancer-specific survival (CSS), disease-free survival (DFS), and recurrence risk (Table 5).

*Fusobacterium nucleatum* was the most consistently investigated organism. In large prospective cohorts, higher intratumoral abundance of *F. nucleatum* was independently associated with increased colorectal-cancer-specific mortality after adjustment for tumor stage and molecular characteristics [32]. In contrast, another prospective tissue-based cohort study did not demonstrate a statistically significant survival difference according to F. nucleatum detection status, highlighting inter-cohort variability [23].

Beyond *F. nucleatum*, other bacterial taxa were also associated with prognosis. In a prospective Chinese cohort, increased intratumoral abundance of *F. nucleatum* and enterotoxigenic *Bacteroides fragilis* was associated with inferior overall survival and disease-free survival in multivariable models [47]. In another study, a specific operational taxonomic unit (OTU_104), classified within the order *Clostridiales*, was independently associated with increased recurrence risk, with each incremental rise in relative abundance corresponding to a higher hazard of disease recurrence [35].

Conversely, not all bacterial species were prognostically relevant. In a large population-based cohort including 1313 patients, the amount of intratumoral *Bifidobacterium* DNA was not significantly associated with colorectal-cancer-specific or overall survival after inverse probability weighting and multivariable adjustment [30].

Some observational studies further reported associations between higher bacterial burden and advanced tumor stage or aggressive histopathological features, indirectly supporting a link between microbial colonization and tumor progression [44]. However, heterogeneity in microbiome detection techniques, cutoff definitions, and covariate adjustment strategies limits direct comparison across studies.

Overall, current evidence suggests that specific tumor-associated microbes—particularly elevated intratumoral *Fusobacterium nucleatum*—may be associated with adverse oncologic outcomes in colorectal cancer [32,47]. Nevertheless, the available data remain observational, and standardized prospective validation is required before microbiome-based biomarkers can be incorporated into established prognostic models.

### 3.3. Influence of Gut Microbiome on Treatment Response in CRC

Two clinical studies evaluated the association between baseline gut microbiome composition and treatment response in colorectal cancer, focusing on systemic therapy and combined neoadjuvant strategies (Table 6).

In a phase Ib/II study of metastatic colorectal cancer patients treated with regorafenib, distinct pretreatment stool microbiome profiles were observed between responders (partial response or stable disease) and patients with progressive disease [46]. Differences in community structure were evident at the beta-diversity level, and specific bacterial taxa were enriched in responders compared to non-responders. Importantly, microbiome composition was associated with progression-free survival in multivariable models, suggesting that baseline microbial signatures may independently correlate with treatment efficacy. These findings indicate that gut microbiota may influence therapeutic benefit in the refractory metastatic setting.

Similarly, in patients with locally advanced rectal cancer receiving combined neoadjuvant chemoradiotherapy and immune checkpoint inhibition, baseline stool microbiome composition was associated with both pathological complete response and the development of treatment-related adverse events [33]. Enrichment of specific bacterial genera, including short-chain fatty acid–producing taxa, was observed in patients demonstrating favorable responses. Although exploratory and limited by sample size, these results support the hypothesis that the gut microbiome may modulate both therapeutic efficacy and toxicity during multimodal treatment.

Collectively, available evidence suggests that baseline gut microbial composition may influence response to systemic therapy and immunochemoradiotherapy in colorectal cancer. However, current data derive from small, heterogeneous cohorts, and the methodologies used for microbiome profiling vary substantially. While these findings are biologically plausible, given the established interactions between the gut microbiota, immune modulation, and drug metabolism, they remain hypothesis-generating. Prospective validation in larger, standardized cohorts is required before microbiome-guided therapeutic stratification can be considered in routine clinical practice.

## 4. Discussion

This systematic review summarizes current clinical evidence on the role of the gut microbiome in colorectal cancer (CRC) diagnosis, prognosis, and treatment response. Despite methodological heterogeneity, consistent patterns of microbial dysbiosis were observed across populations, supporting a biologically relevant association between alterations in the microbiome and colorectal carcinogenesis. Importantly, despite this heterogeneity, consistent microbial patterns across independent cohorts support the robustness of these associations.

Multiple diagnostic studies identified microbial signatures that distinguished CRC patients from healthy controls with moderate-to-high accuracy [18,19,20,21,22,24,26,28,31]. In several cohorts, combining microbial markers with clinical variables or fecal occult blood testing further improved performance [21,28].

While colonoscopy remains the gold standard, its invasive nature and limited adherence highlight the need for complementary strategies. Stool-based microbiome profiling may serve as a non-invasive adjunct for risk stratification and early detection. Notably, microbial shifts have been reported at the adenoma stage [20,24,31], suggesting that dysbiosis may precede overt malignancy. Distinct tumor-associated microbial niches further support the biological plausibility of microbiome-based screening approaches [56].

The rising incidence of early-onset CRC underscores the role of environmental and lifestyle factors [3,34]. Unlike genetic mutations, the gut microbiome is dynamic and potentially modifiable. Diet, obesity, antibiotic exposure, and stress have all been implicated in dysbiosis and CRC development [57]. These position the microbiome as a possible preventive target, particularly in younger populations. Although causality remains to be clarified, its modifiable nature distinguishes it from conventional molecular biomarkers and supports further investigation into microbiome-directed prevention strategies.

Several cohort studies reported associations between intratumoral microbial composition and survival outcomes [23,32,35,47]. Increased abundance of *Fusobacterium nucleatum* has been linked to inferior cancer-specific survival in multivariable models [32], although findings are not entirely consistent across studies [23].

Whether tumor-associated bacteria act as drivers of aggressive disease or selective colonizers of altered tumor microenvironments remains uncertain. Experimental data suggest potential mechanistic links through immune modulation and oncogenic signaling pathways, but current clinical evidence remains largely observational [58,59].

Emerging data indicate that baseline microbiome composition may influence response to systemic therapy [33,46]. Associations between microbial diversity and progression-free survival, as well as between microbial profiles and pathological response, suggest a modulatory role in treatment efficacy. Mechanistically, microbiota may affect immune activation and drug metabolism [58,59]. However, available studies are small and heterogeneous, and microbiome profiling cannot yet guide therapeutic decisions outside research settings.

Substantial variability in sampling methods, sequencing techniques, bioinformatic pipelines, and confounder adjustment limits comparability across studies. Additionally, microbial composition varies by geography and ethnicity [18,25,34], raising concerns regarding generalizability. The integration of microbiome sequencing into clinical practice also requires attention to cost, standardization, and equitable access to avoid widening disparities in CRC care.

Finally, the observational nature of most included studies precludes causal inference, and reverse causality between tumor biology and microbiome composition cannot be excluded.

### Future Directions

Future research should prioritize prospective validation, standardized methodologies, and integration of microbiome data into multi-omics frameworks. Advances in computational modeling and spatial profiling may help clarify causality and strengthen biomarker development. Ultimately, interventional trials will determine whether microbiome modulation can meaningfully improve CRC prevention and outcomes.

## 5. Conclusions

The gut microbiome represents a rapidly evolving field in colorectal cancer research, with increasing evidence supporting its potential clinical relevance. Identifying the significance of certain microbial agents as diagnostic and prognostic biomarkers and as treatment targets can alter how we view colorectal cancer. However, despite promising evidence, integrating microbiome profiling into routine clinical practice requires standardized methodologies, prospective validation, and equitable access to sequencing technologies. A deeper understanding of host–microbiome interactions may ultimately enable more precise, preventive, and personalized strategies in colorectal cancer management.

## Figures and Tables

**Table 1:** Studies included in the synthesis.

First_Author	Year	Country	Study Design	Sample Size Total	CRC Patients	Control	Adenoma Patients	Outcome Definition
Avuthu et al. [20]	2022	Multi-country (training: USA, China, France; validation: Austria, Japan)	Meta-analysis/secondary analysis of public fecal shotgun metagenomic datasets + external validation	957	483	474	0	CRC diagnosis (case vs. control) using microbial biomarkers/classifier performance. A reduced set of 21 species markers validated across cohorts
Bosch et al. [21]	2022	The Netherlands	Prospective multicenter case–control study	53	12	20	21	Diagnostic discrimination between CRC, adenoma, and controls using microbiome and integrated biomarker panels
Bundgaard-Nielsen et al. [22]	2019	Denmark	Retrospective cohort study using archived tissue samples	299	99	104	96	Prognosis (5-year overall survival and disease-free survival) and microbiome association with CRC presence
Dai et al. [23]	2018	Multi-country (China, Europe, USA cohorts)	Meta-analysis of public shotgun metagenomic datasets	526	261	265	0	CRC diagnosis using microbiome-based classifier
Feng et al. [24]	2015	China	Case–control study	156	46	61	49	CRC diagnosis and microbiome changes across adenoma–carcinoma progression
Flanagan et al. [25]	2014	Ireland	Retrospective cohort study	122	99	23	0	CRC prognosis (overall survival and disease outcome) and association with CRC stage
Hester et al. [26]	2015	USA	Case–control study	20	10	10	0	CRC diagnosis and microbiome composition differences
Hibberd et al. [27]	2017	Sweden	Case–control study with probiotic intervention arm	36	15	21	0	CRC diagnosis and microbiome composition differences
Kharofa et al. [28]	2023	Multi-country (meta-analysis of curated Metagenomic Data datasets)	Meta-analysis of shotgun metagenomic datasets	1301	692	609	0	CRC diagnosis and microbiome association with CRC risk, including early-onset CRC
Kinross et al. [29]	2011	UK	Case–control study	35	15	20	0	CRC diagnosis and microbiome association with tumor tissue
Kosumi et al. [30]	2018	USA, Japan (Harvard cohorts: NHS and HPFS)	Prospective cohort study	1313	1313	0	0	CRC prognosis (overall survival and cancer-specific survival)
Leung et al. [31]	2019	Hong Kong and Australia	Case–control study (paired tumor and adjacent normal mucosa)	19	19	0	0	CRC diagnosis and prognosis association with microbiome composition
Li et al. [32]	2022	Multi-country (7 countries including China, Europe, USA)	Multi-cohort meta-analysis of shotgun metagenomic datasets plus in-house validation cohort	2101	748	882	471	CRC diagnosis and microbiome biomarker identification
Liu et al. [33]	2022	Multi-country (China, Japan, Germany, France, Austria, USA, Italy, Australia cohorts)	Multi-cohort meta-analysis of shotgun metagenomic sequencing datasets plus independent validation cohort	1368	724	644	0	CRC diagnosis using microbiome biomarkers
Ma et al. [34]	2024	China	Prospective observational cohort study	32	32	0	0	Treatment response/toxicity: development of adverse events during neoadjuvant chemoradiotherapy combined with immunotherapy
Mima et al. [35]	2016	USA (NHS/HPFS cohorts; tumor specimens)	Prospective cohort (CRC cases within NHS & HPFS) with tumor-tissue molecular analysis	1069	1069	0	0	Prognosis: colorectal-cancer-specific mortality (primary), overall mortality (secondary)
Mima et al. [36]	2015	USA (Nurses’ Health Study and Health Professionals Follow-up Study)	Prospective cohort with tumor molecular and immune profiling	598	598	0	0	CRC prognosis stratified by immune infiltration (CD3+, CD8+, CD45RO+, FOXP3+ T-cell density)
Noguti et al. [37]	2016	USA and Serbia	Prospective multicenter cohort study	91	91	0	0	CRC prognosis (disease-free survival and recurrence risk)
Ogashi et al. [38]	2013	Japan	Case–control study	142	93	27	22	CRC diagnosis and association with microbiome metabolic environment
Osman et al. [39]	2021	Malaysia	Case–control study with validation cohort	96	58	38	0	CRC diagnosis and biomarker development
Piciocchi et al. [40]	2021	Italy, Poland (multicenter European cohort)	Multicenter case–control study	325	29	162	79 adenomas (+55 hyperplastic polyps)	CRC diagnosis and progression risk (adenoma and adenocarcinoma development)
Shah et al. [41]	2017	Multi-country (pooled public datasets; lead group USA)	Meta-analysis of 9 fecal 16S rRNA studies + machine learning classifier development	509	195	235	79	CRC diagnosis (CRC vs. control; adenoma vs. control) using stool microbiome classifier; also evaluated adding clinical markers (FOBT/age/sex/BMI when available)
Shinba et al. [42]	2016	USA	Case–control study	131	42	89	0	CRC diagnosis and microbiome-metabolite associations
Taylor et al. [43]	2020	New Zealand	Observational cohort study (method comparison study)	11	11	0	0	CRC microbiome characterization and sequencing method validation
Viljoen et al. [44]	2015	South Africa	Observational cohort study	73	73	0	0	CRC diagnosis and association with tumor stage and clinicopathological characteristics
Vogtmann et al. [45]	2016	USA	Case–control study with validation cohort	104	52	52	0	CRC diagnosis and microbiome biomarker validation
Wang et al. [46]	2021	China	Phase Ib/II single-arm clinical trial + translational microbiome analysis	42	42	0	0	Treatment response + prognosis: PR/SD vs. PD; PFS/OS; microbiome predictors of PFS
Wei et al. [47]	2016	China	Prospective cohort study	180	180	0	0	CRC prognosis (overall survival and disease-free survival)
Wu et al. [48]	2019	China	Observational cohort study	30	30	0	0	CRC progression and tumor aggressiveness (clinicopathological associations)
Xiang et al. [49]	2023	China/Multi-ethnic GWAS consortia (AGWAS, FinnGen)	Mendelian randomization study using microbiome and CRC GWAS summary statistics	33,970	6692 + 7427 CRC patients (two GWAS cohorts)	27,278 + 25,600 controls	0	CRC risk (causal inference between microbiome and CRC risk)
Xie et al. [50]	2017	China	Case–control study with independent validation cohort	1037 (combined test + validation cohorts)	445	304	288	CRC diagnosis and early detection biomarker performance
Yang et al. [51]	2021	China	Case–control study with independent validation cohort and metagenomic analysis	1038 (728 discovery + 310 validation cohort)	564 CRC (185 young-onset CRC, 379 old-onset CRC)	474	0	CRC diagnosis and microbiome differences between young- and old-onset CRC
Yazici et al. [52]	2017	USA	Case–control study	329	153	176	0	CRC diagnosis and microbiome associations with racial differences and tumor biology
Yu et al. [53]	2015	China, Denmark (validation cohorts)	Case–control study with independent validation cohorts	452 (combined discovery + validation cohorts)	184	268	0	CRC diagnosis and microbiome biomarker development
Zeller et al. [54]	2014	France and Germany	Case–control study with validation cohort	156	53	61	42	CRC diagnosis and biomarker development
Zhang et al. [55]	2018	China	Case–control study	206	67	62	77	CRC diagnosis and microbiome changes during tumor progression

CRC: colorectal cancer, OS: overall survival, DFS: disease-free survival, PFS: progression-free survival, RR: recurrence rate, PR: partial response, SD: stable disease, PD: progressive disease, pCR: pathological complete response, RECIST: Response Evaluation Criteria in Solid Tumors, FOBT: fecal occult blood test, NHS: Nurses’ Health Study, HPFS: Health Professionals Follow-up Study, CD3+: cluster of differentiation 3 positive T lymphocytes, CD8+: cluster of differentiation 8 positive cytotoxic T lymphocytes, CD45RO+: cluster of differentiation 45RO positive memory T lymphocytes, FOXP3+: forkhead box P3 positive regulatory T lymphocytes, GWAS: genome-wide association study, AGWAS: Asian Genome-Wide Association Study, 16S rRNA: 16S ribosomal ribonucleic acid gene sequencing, qPCR: quantitative polymerase chain reaction, BMI: body mass index.

**Table 2:** Patients’ demographics.

Researcher	No of CRC Patients	Age (MEAN)	BMI (MEAN)	Disease Stage
Avuthu et al. [20]	483	61.84–67.06 years (mean range across cohorts)	23.03–26.50 kg/m^2^	NR
Bosch et al. [21]	12	66.25	26.25	NR
Bundgaard-Nielsen et al. [22]	99	70.75	NR	Stage I: 7.1%, Stage II: 41.4%, Stage III: 43.4%, Stage IV: 8.1%
Dai et al. [23]	261	65.93	25.3	Stage I–IV CRC included across cohorts (exact breakdown not reported)
Feng et al. [24]	46	67.07	26.5	Stage 0: 7; Stage I: 17; Stage II: 9; Stage III: 11; Stage IV: 1; Unknown: 1.
Flanagan et al. [25]	99	67	NR	Stage I: 13; Stage II: 72; Stage III: 32; Stage IV: 5.
Hester et al. [26]	10	59.9	32.7	NR
Hibberd et al. [27]	15	76.75	23.88	Stage I: 2; Stage II: 6; Stage III: 7
Kharofa et al. [28]	692	63.25	24.33	NR
Kinross et al. [29]	15	73	28.3	Majority locally advanced tumors (T3–T4).
Kosumi et al. [30]	1313	69.3	NR	Stage I: 306; Stage II: 389; Stage III: 350; Stage IV: 151
Leung et al. [31]	19	64.7	NR	T1: 1; T2: 1; T3: 8; T4: 9. Majority locally advanced CRC.
Li et al. [32]	748	61.8	27.89 (*n* = 523 patients)	Stage I: 139; Stage II: 115; Stage III: 119; Stage IV: 115, Unkown: 28
Liu et al. [33]	724	NA	NA	NA
Ma et al. [34]	32	Not clearly reported	Not clearly reported	Locally advanced rectal cancer (exact TNM stage not reported; patients eligible for neoadjuvant therapy)
Mima et al. [35]	1069	69.3	NR	AJCC: I 241; II 325; III 278; IV 133
Mima et al. [36]	598	67.2	NR	AJCC: I 123; II 191; III 174; IV 77
Noguti et al. [37]	91	67	25.9	Early-stage colon cancer (I & II = 37 III = 48)
Ogashi et al. [38]	93	68.9	22.1	Dukes A: 36; Dukes B: 19; Dukes C: 24; Dukes D: 14
Osman et al. [39]	58	64.88	NR	Dukes A: 2; Dukes B: majority discovery cohort; Dukes C: majority validation cohort; Dukes D included
Piciocchi et al. [40]	29	64	25.9	NR
Shah et al. [41]	195	NR	NR	Not consistently reported across source studies (some had TNM/M stage data; incomplete)
Shinba et al. [42]	42	63.4	25.8	AJCC: I & II 9; III 18; IV 14; Unknown 1
Taylor et al. [43]	11	NR	NR	TNM Stage I: 3; Stage II: 3; Stage III: 5; Stage IV: 0
Viljoen et al. [44]	73	57.9	26.6	Stage I: 6; Stage II: 29; Stage III: 22; Stage IV: 16
Vogtmann et al. [45]	52	63.4	25.8	Invasive non-metastatic: 40.4%; Metastatic: 34.6%;
Wang et al. [46]	42	52.4	23.69	Metastatic CRC (Stage IV; metastatic sites reported: liver 69–100% depending on dose cohort)
Wei et al. [47]	180	62.2	NR	Stage I–III (curative surgery) and Stage IV (palliative surgery) included
Wu et al. [48]	30	NR	NR	Tumor differentiation groups: moderately/poorly differentiated vs. well differentiated
Xiang et al. [49]	6692 + 7427 CRC patients (two GWAS cohorts)	NA	NA	NA
Xie et al. [50]	445	NR	NR	Early CRC: 142; Advanced CRC: 303
Yang et al. [51]	564 CRC (185 young-onset CRC, 379 old-onset CRC)	40.45	NR	Stage 0–IV CRC included (stage-specific analysis performed)
Yazici et al. [52]	153	40.08	28.8	Stage I–IV CRC included; stage distribution similar between racial groups
Yu et al. [53]	184	67	NR	Stage I: 20; Stage II: 45; Stage III: 49; Stage IV: 8 (Chinese cohort); additional stages included in validation cohorts
Zeller et al. [54]	53	NR	NR	Stage I 15; II 7; III 23; IV 21
Zhang et al. [55]	67	60.5	NR	Stage I–IV CRC included; stage-specific analysis performed

CRC = Colorectal cancer; BMI = Body mass index; NR = Not reported; NA = Not applicable; AJCC = American Joint Committee on Cancer; TNM = Tumor–Node–Metastasis classification; T = Primary tumor stage (Tumor size/depth); GWAS = Genome-wide association study; Dukes = Dukes staging classification system; I–IV = Stage 1–4; T1–T4 = Tumor stages 1–4.

**Table 3:** Microbial Biomarkers and Diversity Differences Associated with CRC Diagnosis.

Study (Year)	Sample/Method	Key Microbial Findings in CRC	Alpha Diversity	Beta Diversity	Diagnostic Interpretation
Bosch 2022 [21]	Stool/16S + multi-omics	Selected taxa (e.g., *Methanobrevibacter*, *Bifidobacterium*, *Ruminococcaceae*) via ML panel	No difference	No difference	Diagnostic value driven by taxa panel rather than diversity
Hibberd 2017 [27]	Tissue + stool/16S	↑ *Fusobacterium*, *Peptostreptococcus*, *Selenomonas*	Increased	Distinct clustering	Tumor-associated microbial signature
Bundgaard-Nielsen 2019 [22]	Tissue/qPCR + 16S	↑ *Prevotella* (CRC), ↑ *Acinetobacter* (adenoma), *Fusobacterium* enriched in tumors	Variable	Compositional differences	Microbiome differs across tissue types
Flanagan 2014 [25]	Tissue + stool/qPCR	↑ *Fusobacterium nucleatum* (CRC and adenomas)	Not reported	Not reported	Strong single-species biomarker
Yang 2021 [51]	Stool/16S + metagenomics	↑ *Fusobacterium*, *Streptococcus*, *Gemella*; distinct profiles in EOCRC	Decreased	Significant separation	Distinct CRC microbial signature
Leung 2019 [31]	Tissue/16S + ddPCR	↑ *Fusobacterium*, ↑ *Campylobacter* (tumor)	No difference	Separation present	Tumor-specific microbial profile
Liu 2022 [33]	Stool/shotgun	↑ *F. nucleatum*, *P. micra*, *A. muciniphila*; ↓ butyrate producers	Decreased	Significant separation	Multi-species marker panels most informative
Li 2025 [32]	Stool/shotgun	Archaeal dysbiosis in CRC	Decreased	Significant separation	Archaea contribute to diagnostic signal
Kharofa 2023 [28]	Stool/meta-analysis	Species-level associations; EOCRC-specific interactions	No difference	No clear clustering	Diversity alone not diagnostic
Shah 2018 [41]	Stool/16S (pooled)	Recurrent taxa (*Fusobacterium*, *Peptostreptococcus*, *Dialister*)	Variable	Study-dependent	Composite biomarkers outperform diversity
Zeller 2014 [54]	Stool/shotgun	22-species panel (incl. *Fusobacterium*, *Peptostreptococcus*)	Not primary	Clear separation	Multi-species classifier effective
Yu 2015 [53]	Stool/shotgun	↑ *Parvimonas micra*, ↑ *F. nucleatum*, ↑ *P. stomatis*	Decreased	Significant clustering	Species-level classifier validated

EOCRC: early-onset colorectal cancer, ML: machine learning, qPCR: quantitative polymerase chain reaction. ↑ = increased, ↓ = decreased.

**Table 4:** Summary of studies evaluating the diagnostic value of the gut microbiome in colorectal cancer.

First Author	Sample Type	Method	Key Microbial Findings	Diagnostic Performance	Main Conclusion
Avuthu et al. [20]	Stool	Shotgun	21-species signature	AUC ~0.85–0.90	Multi-species classifier validated across cohorts
Bosch et al. [21]	Stool	16S + multi-omics	Microbiome + proteomics	AUC > 0.90	Integrated models improve discrimination
Dai et al. [23]	Stool	Shotgun	Cross-cohort microbial markers	AUC ~0.84	Reproducible CRC-associated taxa
Liu et al. [33]	Stool	Shotgun	Multi-cohort microbial signature	AUC ~0.90	Strong classifier across populations
Osman et al. [39]	Tissue + Stool	16S + qPCR	Fn, Pm, Ps panel	AUC ~0.85	Multi-bacteria panel effective
Shah et al. [41]	Stool	16S	Composite microbial biomarker	AUC ~0.80	Improved with FOBT combination
Zeller et al. [54]	Stool	Shotgun	Microbial gene markers	AUC ~0.84	Early detection potential
Yu et al. [53]	Stool	Shotgun	CRC-associated species	AUC ~0.90	Validated across cohorts

Fn = Fusobacterium nucleatum, Pm = Parvimonas micra, Ps = Peptostreptococcus stomatis.

**Table 5:** Studies evaluating the association between microbiome composition and colorectal cancer prognosis.

First Author	Sample Type	Method	Key Microbial Findings	Outcome	Main Conclusion
Mima et al. [35]	Tumor tissue	qPCR	High Fn abundance	CSS, OS	Associated with worse survival
Wei et al. [47]	Tumor tissue	16S	Fn + ETBF enrichment	OS, DFS	Poor prognosis correlation
Noguti et al. [37]	Tumor tissue	16S	Specific OTUs	DFS, recurrence	Microbiome linked to recurrence risk
Kosumi et al. [30]	Tumor tissue	qPCR	Bifidobacterium	OS	No significant association
Flanagan et al. [25]	Tumor tissue	qPCR	Fn presence	Survival	Associated with disease progression

Fn: *Fusobacterium nucleatum*, ETBF: enterotoxigenic *Bacteroides fragilis*, OS: overall survival, CSS: colorectal-cancer-specific survival, DFS: disease-free survival, 16S rRNA: 16S ribosomal RNA gene sequencing.

**Table 6:** Gut Microbiome and Treatment Response in CRC.

Study [Ref]	Clinical Setting	Treatment Regimen	Microbial Findings Associated with Response	Diversity Findings	Clinical Endpoint	Independent Predictor?	Clinical Interpretation
Ma et al. [33]	Locally advanced rectal cancer (MSS/pMMR)	Neoadjuvant chemoradiotherapy + immune checkpoint inhibitor	Differential baseline abundance of specific genera associated with development of treatment-related adverse events; enrichment of short-chain fatty acid–producing bacteria in responders	Distinct baseline microbial composition between patients with and without grade 1–2 adverse events	Pathologic complete response (pCR); treatment-related toxicity	Exploratory (small cohort)	Baseline gut microbiome may influence both therapeutic efficacy and toxicity during combined neoadjuvant immunochemoradiotherapy
Wang et al. [46]	Metastatic CRC	Regorafenib (Phase Ib/II trial)	Higher relative abundance of certain commensal taxa in patients with partial response (PR) or stable disease (SD); enrichment of pro-inflammatory/pathogenic taxa in progressive disease (PD)	Differences in beta-diversity between PR/SD vs. PD groups; altered community structure associated with survival	PFS and OS	Yes (multivariable analysis for PFS)	Pretreatment microbiome composition may stratify likelihood of benefit from regorafenib and correlate with survival outcomes

PR: partial response, SD: stable disease, PD: progressive disease, PFS: progression-free survival, OS: overall survival, pCR: pathologic complete response, MSS: microsatellite stable, pMMR: proficient mismatch repair, 16S rRNA: 16S ribosomal RNA gene sequencing, SCFA: short-chain fatty acids, CI: confidence interval.

## Data Availability

All data is available upon request from the corresponding author.

## Supplementary Materials

The following supporting information can be downloaded at: https://www.mdpi.com/article/10.3390/medicina62061050/s1, Table S1. Risk of bias assessment of included studies. Table S2. DNA extraction and sequencing details.

## Abbreviations

AJCC	American Joint Committee on Cancer
Am	*Akkermansia muciniphila*
ASVs	amplicon sequence variants
AUC	area under the curve
AUROC	area under the receiver operating characteristic curve
BRAF	v-Raf murine sarcoma viral oncogene homolog B
Bray–Curtis	Bray–Curtis dissimilarity index
CIMP	CpG island methylator phenotype
CI	confidence interval
CRC	colorectal cancer
CSS	Colorectal-cancer-specific survival
ddPCR	droplet digital polymerase chain reaction
DFS	disease-free survival
dMMR	deficient mismatch repair
DNA	deoxyribonucleic acid
EOCRC	early-onset colorectal cancer
ETBF	enterotoxigenic *Bacteroides fragilis*
FFPE	formalin-fixed paraffin-embedded
FIT	fecal immunochemical test
Fn	*Fusobacterium nucleatum*
FOBT	fecal occult blood test
FPR	false positive rate
GWAS	genome-wide association study
HPLC	high-performance liquid chromatography
HR	hazard ratio
LC-MS/MS	liquid chromatography–tandem mass spectrometry
LINE-1	long interspersed nucleotide element-1
ML	machine learning
MR	Mendelian randomization
MSI	microsatellite instability
MSS	microsatellite stable
OTU	operational taxonomic unit
OR	odds ratio
OS	overall survival
PCA	principal component analysis
PCoA	principal coordinates analysis
PD	progressive disease
PD-L1	programmed death-ligand 1
PERMANOVA	permutational multivariate analysis of variance
PFS	progression-free survival
pCR	pathologic complete response
pMMR	proficient mismatch repair
Pm	*Parvimonas micra*
POD model	probability of disease model
PR	partial response
Ps	*Peptostreptococcus stomatis*
qPCR	quantitative polymerase chain reaction
RDA	redundancy analysis
RECIST	Response Evaluation Criteria in Solid Tumors
ROC	receiver operating characteristic
RR	recurrence rate
SCFA	short-chain fatty acids
SD	stable disease
TNM	tumor–node–metastasis staging system
UniFrac	unique fraction phylogenetic distance metric
Wnt	Wingless-related integration site signaling pathway
yCRC	young-onset colorectal cancer
