# Supplementary material for: Impact of Gut Microbiota on the Clinical Course and Treatment Outcomes of Colorectal Cancer—A Systematic Review
Source: Medicina (Kaunas). 2026 May 28;62(6):1050. doi: 10.3390/medicina62061050 (PMC13303677; doi:10.3390/medicina62061050)
Supplement: Supplementary file 1 [file medicina-62-01050-s001.zip › medicina-4205707-supplementary.pdf]

Supplementary Table S1. Risk of bias assessment of included studies.

First Author (Year)	Study Design	Tool Used	Selection Bias	Comparability	Outcome Assessment	Overall Risk of Bias
Avuthu et al. [20]	Meta-analysis	NOS	Low	Moderate	Low	Moderate
Bosch et al. [21]	Case–control	NOS	Low	Moderate	Low	Moderate
Bundgaard-Nielsen et al. [22]	Retrospective cohort	NOS	Moderate	Moderate	Moderate	Moderate
Dai et al. [23]	Meta-analysis	NOS	Low	Moderate	Low	Moderate
Feng et al. [24]	Case–control	NOS	Low	Moderate	Low	Moderate
Flanagan et al. [25]	Retrospective cohort	NOS	Moderate	Low	Moderate	Moderate
Hibberd et al. [27]	Case–control	NOS	Moderate	Low	Moderate	Moderate
Khrofa et al. [28]	Meta-analysis	NOS	Low	Moderate	Low	Moderate
Kosumi et al. [30]	Prospective cohort	NOS	Low	High	Low	Low
Leung et al. [31]	Case–control	NOS	Moderate	Moderate	Moderate	Moderate
Li et al. [32]	Meta-analysis	NOS	Low	Moderate	Low	Moderate
Liu et al. [33]	Meta-analysis	NOS	Low	Moderate	Low	Moderate
Ma et al. [34]	Prospective cohort	ROBINS-I	Moderate	Moderate	Moderate	Moderate
Mima et al. [35]	Prospective cohort	NOS	Low	High	Low	Low
Noguti et al. [37]	Prospective cohort	NOS	Moderate	Moderate	Moderate	Moderate
Osman et al. [39]	Case–control	NOS	Moderate	Moderate	Moderate	Moderate
Shah et al. [41]	Meta-analysis	NOS	Low	Moderate	Low	Moderate

First Author (Year)	Study Design	Tool Used	Selection Bias	Comparability	Outcome Assessment	Overall Risk of Bias
Vogtmann et al. [45]	Case–control	NOS	Moderate	Moderate	Moderate	Moderate
Wang et al. [46]	Clinical trial	ROBINS-I	Moderate	Moderate	Moderate	Moderate
Wei et al. [47]	Prospective cohort	NOS	Moderate	Moderate	Moderate	Moderate

NOS: Newcastle–Ottawa Scale; ROBINS-I: Risk Of Bias In Non-randomized Studies of Interventions

Supplementary Table S2. DNA extraction and sequencing details.

First author	Sample Type	Sequencing Method	Sequencing Platform	16S Region	Bioinformatics Pipeline	Reference Database
**Avuthu et al. [20]**	Stool (fecal)	Shotgun metagenomic sequencing (secondary analysis of existing datasets)	platform varies by original datasets	NA	QC + host/PhiX removal: FastQC, BBduk, BBmap; taxonomic profiling: MetaPhlAn2; downstream: phyloseq + vegan (alpha/beta estimation); feature selection: LEfSe + Random Forest + co-occurrence networks (DyNet); functional/strain-level: PanPhlAn; metabolite links using Japanese metabolomics from original Yachida study	MetaPhlAn2 marker gene reference (~17k genomes; clade-specific markers)

**Bosch et al. [21]**	Stool (fecal)	16S rRNA gene sequencing	Illumina MiSeq (paired-end 2×250 bp)	V4 region (515F–806R primers)	QIIME2; LEfSe; Elastic Net; LASSO; logistic regression models	SILVA-132-99% OTUs database
**Bundgaard-Nielsen et al. [22]**	Formalin-fixed paraffin-embedded colorectal tissue (tumor tissue, paired normal mucosa, adenomas, diverticula)	qPCR for targeted bacteria and 16S rRNA gene sequencing	Illumina MiSeq	V4 region (515F–806R primers)	FastQC for quality control; standard Illumina microbiome processing pipeline	Not explicitly specified (standard 16S reference databases assumed)
**Dai et al. [23]**	Stool (fecal)	Shotgun metagenomic sequencing	Illumina sequencing platforms (HiSeq/MiSeq depending on cohort)	NA	MetaPhlAn taxonomic profiling; Random Forest classification model; cross-validation and external validation across cohorts	MetaPhlAn reference genome database
**Feng et al. [24]**	Stool (fecal)	Shotgun metagenomic sequencing	Illumina HiSeq platform	NA (shotgun sequencing)	MetaPhlAn taxonomic profiling; metagenomic gene analysis; random forest classification models	MetaPhlAn microbial genome database
**Flanagan et al. [25]**	Tumor tissue (fresh frozen and FFPE),	qPCR targeting Fusobacterium nucleatum 16S rRNA gene	ABI Prism real-time PCR system	Targeted 16S rRNA gene	Comparative Ct method for relative quantification	NCBI 16S reference sequence for

	matched normal mucosa			(species-specific primers)		Fusobacterium nucleatum
**Hester et al. [26]**	Stool (fecal)	16S rRNA gene sequencing	Illumina MiSeq	V4 region	QIIME pipeline	Greengenes database
**Hibberd et al. [27]**	Colon mucosal biopsies and stool samples	16S rRNA gene sequencing	Illumina MiSeq	Not explicitly stated (standard 16S region sequencing)	QIIME v1.9.1; uclust OTU clustering (97% similarity); PyNAST alignment; FastTree phylogeny	Greengenes database v13.8
**Khrofa et al. [28]**	Stool (fecal)	Shotgun metagenomic sequencing	Illumina platforms (varies by cohort)	NA	curatedMetagenomicData pipeline; UniRef90 gene annotation; MetaPhlAn taxonomic profiling; mixed-effects regression modeling	UniRef90 gene families; MetaPhlAn reference genomes
**Kinross et al. [29]**	Colon mucosal tissue biopsies	16S rRNA gene sequencing	454 pyrosequencing platform	V3–V5 region	QIIME pipeline; OTU clustering at 97% similarity; phylogenetic analysis	Greengenes reference database
**Kosumi et al. [30]**	Tumor tissue (FFPE specimens)	qPCR quantification of bacterial DNA	ABI real-time PCR platform	Targeted genus-specific assay	Quantitative PCR analysis pipeline	NCBI reference sequences
**Leung et al. [31]**	Tumor tissue and matched adjacent	16S rRNA sequencing	Illumina MiSeq	V3–V4 region	QIIME pipeline; OTU clustering; taxonomic assignment	SILVA reference database

	normal mucosa			(341F–806R)		
**Li et al. [32]**	Stool (fecal)	Shotgun metagenomic sequencing	Illumina platforms	NA	Kraken2 taxonomic classification; Bracken abundance estimation; Random Forest classifier	Genome Taxonomy Database (GTDB)
**Liu et al. [33]**	Stool (fecal)	Shotgun metagenomic sequencing	Illumina platforms (HiSeq, NovaSeq depending on cohort)	NA	Kraken2 taxonomic classification; Bracken abundance estimation; MMUPHin batch correction; Random Forest classifier; PERMANOVA beta diversity analysis	Genome Taxonomy Database (GTDB); UniRef90 gene annotation
**Ma et al. [34]**	Stool (fecal)	Shotgun metagenomic sequencing	Illumina platform	NA	Wilcoxon test, LEfSe analysis, Bray–Curtis distance, PCoA analysis, Random Forest modeling	Not explicitly stated (standard metagenomic reference databases used)
**Mima et al. [35]**	Tumor tissue (FFPE)	Targeted qPCR for *F. nucleatum* DNA normalized to human reference (SLCO2A1; 2^-ΔCt)	ABI real-time PCR (qPCR platform)	NA	qPCR quantification (Ct-based; duplicate runs); Cox proportional hazards models	NA (targeted assay)
**Mima et al. [36]**	Tumor tissue (FFPE)	Targeted qPCR quantification of *Fusobacterium nucleatum* DNA	ABI real-time PCR	NA	Ct-based qPCR quantification; multivariable Cox regression; immune cell quantification via immunohistochemistry	NA (targeted assay)
**Noguti et al. [37]**	Tumor tissue microbiome	16S rRNA gene sequencing	Illumina MiSeq	V4 region	QIIME pipeline; UniFrac distance; Shannon and Pielou	Greengenes database

					diversity indices; OTU-level analysis	
**Ogashi et al. [38]**	Stool (fecal)	Quantitative PCR targeting bacterial 16S/23S rRNA genes	ABI real-time PCR	Targeted bacterial groups (not full 16S sequencing)	Quantitative PCR-based bacterial quantification	Target-specific primers (published validated primers)
**Osman et al. [39]**	Tumor tissue and adjacent mucosal tissue	16S rRNA sequencing (discovery cohort) and species-specific quantitative PCR (validation cohort)	Illumina MiSeq (discovery); ABI real-time PCR (validation)	V3–V4 region	QIIME pipeline; OTU clustering; differential abundance analysis; ROC curve analysis	Greengenes database
**Piciocchi et al. [40]**	Colorectal tissue biopsy samples	Targeted real-time PCR detection of bacterial toxin genes	ABI real-time PCR platform	NA	PCR detection of toxin genes (cif, cdt, cnf1, pks+, bft); multivariate logistic regression analysis	Validated toxin-specific primers
**Shah et al. [41]**	Stool (fecal)	16S rRNA amplicon sequencing (re-analysis of existing datasets)	Mixed: 454-FLX/454-Titanium and Illumina MiSeq across studies	Mixed (V1–V3, V3, V3–V4, V4 across studies)	Two pipelines: QIIME closed reference (QIIME-CR) and StrainSelect+UPARSE (SS-UP)	QIIME-CR: Greengenes; SS-UP: StrainSelect database + UPARSE clustering
**Shinba et al. [42]**	Stool (fecal samples)	16S rRNA gene sequencing and metabolomics analysis	454 pyrosequencing platform	V3–V4 region	OTU clustering; multivariable logistic regression analysis; correlation analysis	Greengenes database
**Taylor et al. [43]**	Tumor tissue	16S rRNA sequencing, MinION nanopore sequencing, and RNA	Illumina MiSeq; Oxford Nanopore MinION; Illumina RNA-seq platform	V3–V4 region (amplicon sequencing)	Kraken2 taxonomic classification; reference genome-based taxonomic assignment	RefSeq genome database

		metatranscriptomic sequencing				
**Viljoen et al. [44]**	Tumor tissue and matched adjacent normal mucosa	Targeted quantitative PCR of bacterial species	ABI real-time PCR platform	NA	qPCR quantification of bacterial abundance; statistical association analysis	Validated species-specific primers
**Vogtmann et al. [45]**	Stool (fecal)	Whole-genome shotgun sequencing and prior 16S rRNA sequencing comparison	Illumina HiSeq shotgun sequencing platform	NA	MetaPhlAn taxonomic profiling; logistic regression; diversity analysis	MetaPhlAn reference genome database
**Wang et al. [46]**	Stool (fecal)	16S rRNA amplicon sequencing	Illumina HiSeq	V3–V4	USEARCH v11 (QC, OTU clustering, taxonomy)	SILVA rRNA database release 132 (RDP classifier)
**Wei et al. [47]**	Tumor tissue microbiome	16S rRNA gene sequencing	NR	NR	OTU clustering (97% similarity), diversity analysis, Cox regression, Kaplan–Meier survival analysis	NR
**Wu et al. [48]**	Tumor tissue	16S rRNA gene sequencing	Illumina high-throughput sequencing	NR	OTU clustering, diversity analysis, differential abundance analysis	NR
**Xiang et al. [49]**	NA	NA	NA	NA	IVW, MR-Egger, weighted median MR methods	GWAS microbiome summary datasets
**Xie et al. [50]**	Stool (fecal)	Quantitative PCR targeting bacterial 16S rRNA genes	ABI real-time PCR	Targeted qPCR (not full 16S sequencing)	Species-specific qPCR quantification	Validated species-specific primers

**Yang et al. [51]**	Stool (fecal)	16S rRNA gene sequencing and metagenomic sequencing	Illumina high-throughput sequencing platform	NR	OTU/ASV clustering, LEfSe analysis, Random Forest classifier, PCoA analysis	NR
**Yazici et al. [52]**	Colon mucosal biopsy (uninvolved mucosa ~10 cm from tumor)	16S rRNA gene sequencing and qPCR for sulfidogenic genes	Illumina MiSeq	NR	OTU clustering, LEfSe, diversity analysis	NR
**Yu et al. [53]**	Stool (fecal)	Whole-genome shotgun metagenomic sequencing and qPCR validation	Illumina MiSeq	NA	Metagenomic gene profiling; mOTU species clustering; gene marker selection; PERMANOVA analysis; random forest classifier	Integrated microbial genome reference catalog
**Zeller et al. [54]**	Stool (fecal)	Whole-genome shotgun metagenomic sequencing	Illumina MiSeq	NA	mOTU species profiling, gene marker analysis, logistic regression classifier	Integrated microbial genome catalog
**Zhang et al. [55]**	Stool (fecal)	16S rRNA gene sequencing	Illumina MiSeq	V3–V4 region	OTU clustering, LEfSe analysis, diversity analysis, PCoA	Greengenes database

**16S rRNA = 16S ribosomal ribonucleic acid; 23S rRNA = 23S ribosomal ribonucleic acid; qPCR = Quantitative polymerase chain reaction; PCR = Polymerase chain reaction; FFPE = Formalin-fixed paraffin-embedded; QC = Quality control; PhiX = PhiX control library (Illumina spike-in control); OTU = Operational taxonomic unit; ASV = Amplicon sequence variant; Ct = Cycle threshold; ΔCt = Delta cycle threshold; ROC = Receiver operating characteristic; IVW = Inverse variance weighted; MR = Mendelian randomization; MR-Egger = Mendelian randomization Egger regression; GWAS = Genome-wide association study; LEfSe = Linear discriminant analysis effect size; PCoA = Principal coordinates analysis; PERMANOVA = Permutational multivariate analysis of variance; UniFrac = Unique fraction metric (phylogenetic distance measure);*

Bray–Curtis = Bray–Curtis dissimilarity index; RDP = Ribosomal Database Project; V1–V5 = Variable regions 1–5 of 16S rRNA gene; MiSeq = Illumina MiSeq sequencing platform; HiSeq = Illumina HiSeq sequencing platform; NovaSeq = Illumina NovaSeq sequencing platform; MinION = Oxford Nanopore MinION sequencing platform; RNA-seq = RNA sequencing; MetaPhlAn = Metagenomic Phylogenetic Analysis; PanPhlAn = Pangenome-based Phylogenetic Analysis; Kraken2 = K-mer–based taxonomic classification tool; Bracken = Bayesian re-estimation of abundance after classification with Kraken; mOTU = Marker gene-based operational taxonomic unit; MMUPHin = Meta-analysis Methods with a Uniform Pipeline for Heterogeneity in microbiome studies; UniRef90 = UniProt Reference Clusters (90% sequence identity); GTDB = Genome Taxonomy Database; RefSeq = NCBI Reference Sequence Database; QIIME = Quantitative Insights Into Microbial Ecology; USEARCH = Usearch sequence analysis tool; UPARSE = Uparse OTU clustering algorithm; QIIME-CR = QIIME Closed Reference pipeline; SS-UP = StrainSelect plus UPARSE pipeline; DyNet = Dynamic Network analysis tool; SLCO2A1 = Solute Carrier Organic Anion Transporter Family Member 2A1 (human reference gene); cif = Cycle inhibiting factor gene; cdt = Cytolethal distending toxin gene; cnf1 = Cytotoxic necrotizing factor 1 gene; pks+ = Polyketide synthase island–positive strain; bft = Bacteroides fragilis toxin gene; ABI = Applied Biosystems real-time PCR platform; NR = Not reported; NA = Not applicable
